# Safety of Simultaneous Scalp and Intracranial EEG and fMRI: Evaluation of RF-Induced Heating

**DOI:** 10.3390/bioengineering12060564

**Published:** 2025-05-24

**Authors:** Hassan B. Hawsawi, Anastasia Papadaki, Vejay N. Vakharia, John S. Thornton, David W. Carmichael, Suchit Kumar, Louis Lemieux

**Affiliations:** 1 Department of Clinical and Experimental Epilepsy, UCL Queen Square Institute of Neurology, University College London, London WC1N 3BG, UK; hassan.hawsawi.15@ucl.ac.uk (H.B.H.); suchit.kumar@ucl.ac.uk (S.K.); 2 MRI Unit, Epilepsy Society, Chalfont St. Peter, Buckinghamshire SL9 0RJ, UK; 3 Administration of Medical Physics, King Abdullah Medical City (KAMC), Makkah 24246, Saudi Arabia; 4 Lysholm Department of Neuroradiology, National Hospital for Neurology and Neurosurgery, UCLH NHS Foundation Trust, Queen Square, London WC1N 3BG, UK; a.papadaki@ucl.ac.uk (A.P.); john.thornton@ucl.ac.uk (J.S.T.); 5 Department of Brain Repair and Rehabilitation, UCL Queen Square Institute of Neurology, University College London, London WC1N 3BG, UK; 6 Department of Clinical and Experimental Epilepsy, National Hospital for Neurology and Neurosurgery, Queen Square, London WC1N 3BG, UK; v.vakharia@ucl.ac.uk; 7 Developmental Imaging and Biophysics Section, UCL Great Ormond Street Institute of Child Health, London WC1N 1EH, UK; david.carmichael@kcl.ac.uk; 8 Wellcome EPSRC Centre for Medical Engineering, King’s College London, St. Thomas’ Hospital, London SE1 7EH, UK

**Keywords:** scalp, electroencephalography, EEG, intracranial, intracranial EEG, icEEG, scalp EEG, magnetic resonance imaging, functional MRI, EEG fMRI, safety, radio frequency, RF heating, temperature measurements

## Abstract

The acquisition of electroencephalography (EEG) concurrently with functional magnetic resonance imaging (fMRI) requires a careful consideration of the health hazards resulting from interactions between the scanner’s electromagnetic fields and EEG recording equipment. The primary safety concern is excessive RF-induced heating of the tissue in the vicinity of electrodes. We have previously demonstrated that concurrent intracranial EEG (icEEG) and fMRI data acquisitions (icEEG-fMRI) can be performed with acceptable risk in specific conditions using a head RF transmit coil. Here, we estimate the potential additional heating associated with the addition of scalp EEG electrodes using a body transmit RF coil. In this study, electrodes were placed in clinically realistic positions on a phantom in two configurations: (1) icEEG electrodes only, and (2) following the addition of subdermal scalp electrodes. Heating was measured during MRI scans using a body transmit coil with a high specific absorption rate (SAR), TSE (turbo spin echo), and low SAR gradient-echo EPI (echo-planar imaging) sequences. During the application of the high-SAR sequence, the maximum temperature change for the intracranial electrodes was +2.8 °C. The addition of the subdural scalp EEG electrodes resulted in a maximum temperature change for the intracranial electrodes of 2.1 °C and +0.6 °C across the scalp electrodes. For the low-SAR sequence, the maximum temperature increase across all intracranial and scalp electrodes was +0.7 °C; in this condition, the temperature increases around the intracranial electrodes were below the detection level. Therefore, in the experimental conditions (MRI scanner, electrode, and wire configurations) used at our centre for icEEG-fMRI, adding six scalp EEG electrodes did not result in significant additional localised RF-induced heating compared to the model using icEEG electrodes only.

## 1. Introduction

The study of brain activity benefits from the integration of data from multiple types of recording, such as EEG, MEG, and functional MRI. For spontaneous brain activity, this can be best achieved using concurrent multimodal acquisitions, as this ensures that all data relates to the same events or brain states. Taking the example of identifying the generators of epileptic activity in patients with severe epilepsy, the localisation of the seizure onset zone with the use of non-invasive techniques such as scalp electroencephalography (EEG) or magnetoencephalography (MEG) is crucial for guiding putative surgical treatment. Given the scalp EEG’s limited capability to detect deep epileptogenic sources [1,2,3] and poor localisation of epileptogenic areas during seizures [4], invasive intracranial EEG (icEEG) is sometimes performed [5,6], using subdural grids and/or depth electrodes. While icEEG provides exquisite sensitivity, this is limited to the electrodes’ immediate vicinity (in particular for depth electrodes) and sparse spatial sampling due to surgical considerations. Relatively rare studies with simultaneous scalp and icEEG recordings show significant differences in discharge morphology and sensitivities [4,7,8,9,10,11,12], as confirmed by computational simulations [13]. For example, the computer simulations of epileptic sources showed that the combined use of scalp EEG and icEEG can improve localisation, albeit this depends on their relative positions [14,15]. Simultaneous scalp EEG, icEEG, and MEG recordings showed better specificity and sensitivity than when performing each modality alone [16]. Furthermore, the combination of functional MRI to EEG (so-called ‘EEG-fMRI’) has provided an entirely new window on epileptic activity in the form of maps of the haemodynamic changes correlated with inter-ictal and seizure discharges on EEG, which up to now has been recorded concurrently with fMRI either on the scalp or intracranially [17,18,19]. We now propose the technique of simultaneous scalp EEG and icEEG fMRI (S-icEEG-fMRI) as a way to overcome some of their individual limitations and improve our understanding of the sources of their respective signals. In order to achieve this, we must first evaluate any potential associated additional health risks.

Scalp and icEEG have been tested individually to determine the associated risks of recording during MRI. At clinical field strengths, radio-frequency (RF)-induced heating of the tissue in the vicinity of EEG electrodes and leads has been found to be the most important safety hazard [20]. Scalp EEG-fMRI has become widely used in neuroscience [17] with the use of special EEG recording equipment. The safety of scalp EEG during MRI has been investigated at 1.5 T, 3 T, and 7 T, leading to a number of recommendations that have allowed scalp EEG recordings during fMRI to be performed with minimal additional risks to the subjects [20,21,22,23,24,25,26,27,28,29,30,31,32]. Concurrent icEEG-fMRI is a more recent development [33,34,35]; moreover, it has been the subject of safety tests [36,37,38,39,40,41], which demonstrated that it can be performed with an acceptable level of additional risk, with the main precautions being controlling the length and position of the leads and limiting protocols to low-specific absorption rate (SAR) sequences and the type of RF coil [6,42]. We recently extended our phantom-based work to demonstrate that the use of the body transmit coil does not necessarily lead to excessive heating [41]. Furthermore, our analyses of brain tissue in the immediate vicinity of implanted depth electrodes exposed to MRI for icEEG-fMRI have demonstrated alterations that are unremarkable compared to tissue (also from the sites of electrode implantation) in brains that were not subjected to MRI [43].

The possibility of implementing concurrent scalp and icEEG recordings during fMRI for the study of epileptic activity in humans requires us to re-evaluate those risks. We also need to consider the type of scalp electrode to be used, given the wide variety of scalp EEG electrodes used clinically, from cups (rings) made of Ag/AgCl, conductive plastic, or gold to subdermal wires. Subdermal wires are used mostly in the intensive care unit for recording EEG signals without the need of re-fixing or re-gelling (as for the more standard cup electrodes) for more than six days [44,45] and up to sixty consecutive days [46]. In addition, during MRI scanning, these electrodes showed few artefacts due to their size [46]. The safety of subdermal needle EEG electrodes and their leads has been investigated on a phantom and a human subject using TSE, FLAIR, and T1 MPRAGE and showed lower heating compared to stainless steel electrodes [47]. Therefore, we propose to utilise subdermal wires for scalp EEG recording combined with icEEG during fMRI scanning. The aim of this work is to determine whether this combination results in significant additional health hazards, using a test phantom.

Previous studies on the safety of RF-induced heating near cerebral electrodes have used various phantoms, ranging from a pair of bags filled with saline [36] to an acrylic spherical head placed next to a torso-shaped container [38] and a combined acrylic square-shaped head and torso (ASTM; [6,37,39,42,48,49,50,51,52,53,54]) (see [20] for a review). For this work, we constructed a new phantom that consists of two elements: First, a realistically shaped head phantom made of a shell designed to hold icEEG electrodes (depth, and grids and strips) along with the necessary conducting gel and scalp EEG electrodes on its outer surface; and second, an acrylic torso model (ASTM) for RF body coil loading.

In the following section, we describe the results of tests specifically aimed at assessing the impact of adding scalp subdermal electrodes to intracranial electrodes on RF-induced heating in their vicinity, using the body transmit coil. Our approach is based on building directly on our previous RF-induced heating tests for icEEG-fMRI [41] to assess the incremental effects of adding scalp electrodes. Therefore, we first conducted heating measurements in the vicinity of a set of icEEG electrodes implanted in the head part of the phantom, providing us with a set of baseline measurements and an opportunity to confirm previous measurements [6,42]. In a second step, we investigated the effect on heating patterns caused by adding a set of scalp subdermal EEG electrodes to the phantom with the implanted icEEG electrodes.

## 2. Methods

In this study, we evaluated heating in the vicinity of EEG electrodes using the body RF coil for transmission, and we conducted two experiments: *Experiment 1* consisted of temperature measurements on a phantom with realistic head geometry with icEEG electrodes placed inside the phantom. To evaluate potential additional risks from the addition of scalp EEG electrodes, we performed *Experiment 2* with repeated temperature measurements on the same phantom and icEEG electrode configuration with additional scalp electrodes on the head phantom’s external surface.

### 2.1. Combined QS-ASTM-II and Realistically Shaped Head Phantom

**Figure 1:**
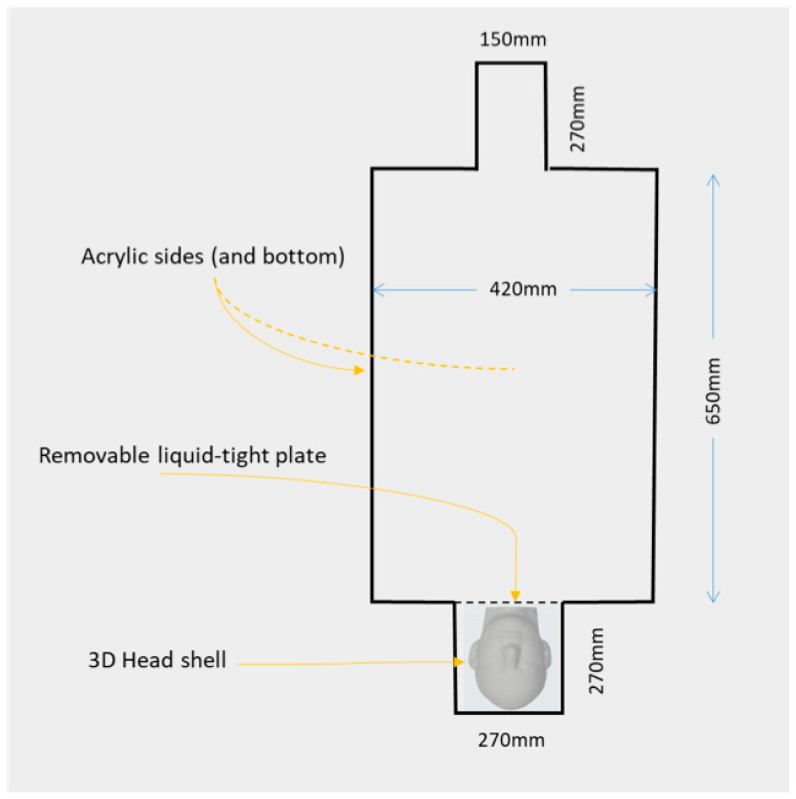
**Schematic representation of the phantom**. The phantom consists of two elements, both of our manufacture and to be filled with an appropriate conducting gel: an acrylic phantom derived from the ASTM design, called QS-ASTM-II, and a realistically shaped 3D head. The realistically shaped head element was placed in the QS-ASTM-II phantom’s head section (which was devoid of gel) and is separated from the torso part by a removable acrylic plate.

The phantom used in this experiment comprised two elements: a box-shaped torso and a realistically shaped head (see Figure 1 for illustration and dimensions). The torso element consists of the main part of a new custom-built acrylic container called *QS-ASTM-II*. The shape and dimensions of QS-ASTM-II are partly based on the standard ASTM phantom designed for heating testing [55], with its main torso part and a ‘head’ part, to which we added a second larger box-shaped ‘head’ part at the ‘feet’ end. The larger head part is designed to contain the realistically shaped head phantom.

The torso part of the phantom was filled with a water-based gel made by mixing 19 litres of distilled water, 8 g/L of polyacrylic acid (PAA) (Sigma–Aldrich, Burlington, MA, USA), and 0.7 g/L of NaCl [6,55,56]. An acrylic partition was used to separate the torso from the part of the QS-ASTM-II phantom containing the realistically shaped head, thereby preventing the torso gel from going around the latter.

**Figure 2:**
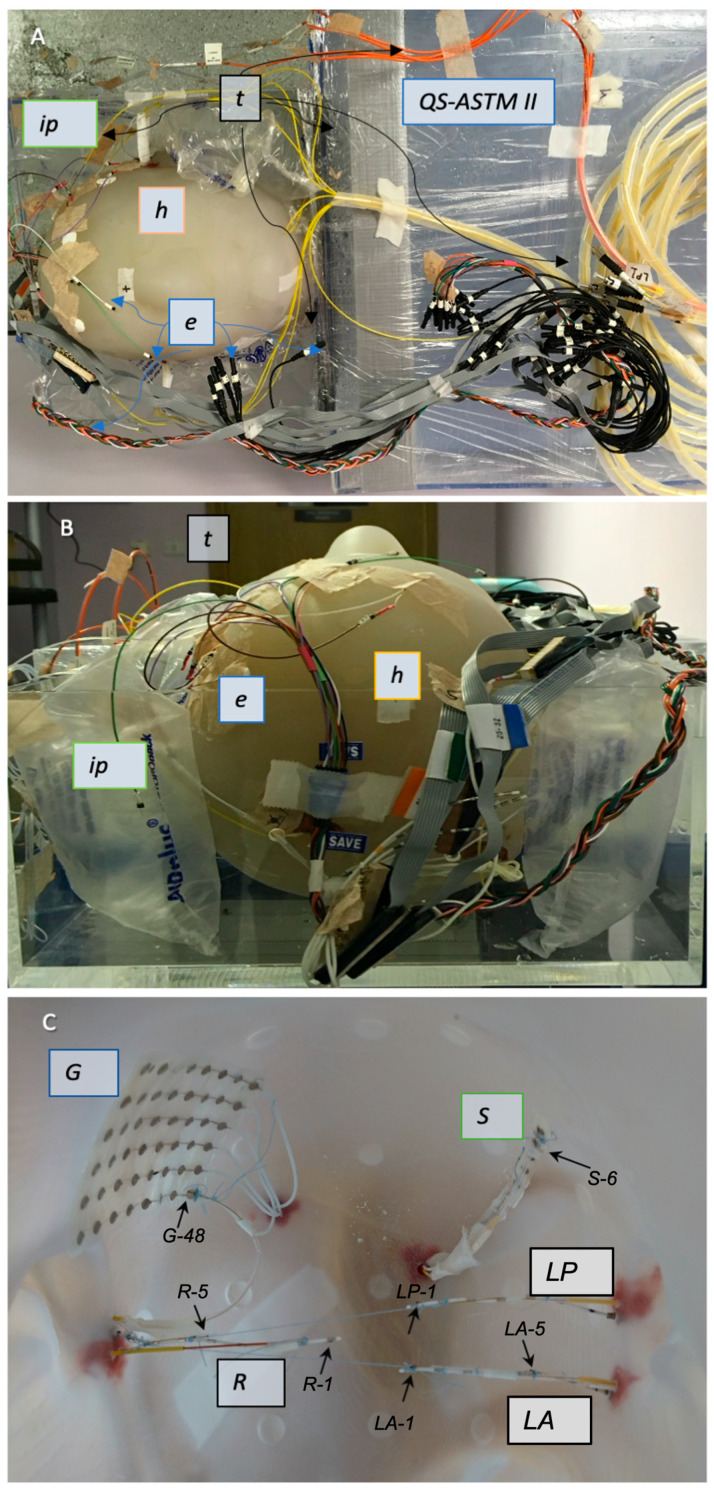
**Experimental setup: phantom with subdermal scalp and icEEG electrodes in place**: (**A**): Frontal view of the QS ASTM II phantom with the head shell (h), the temperature probes (t), the icEEG and Ives electrode wires (e), and the immobilisation pads (ip). (**B**): Axial view of the same setup. (**C**): Internal view of the head shell showing the icEEG electrodes, namely, 3 depth electrodes (with contacts LP-1, LA-1, LA-5, S-6 and G-48 highlighted by small black arrows): right temporal anterior (‘R’), left temporal posterior (‘LP’), and left temporal anterior (‘LA’); a subdural strip over the left parietal–frontal convexity (at the superior region of the head) (‘S’); and a right frontal subdural grid (‘G’). See Figure 3 for a visualisation of the electrodes inside the phantom.

The head part of the phantom was made based on a 3D computer model derived from the CT scan of a human adult male that was exported as a surface render. It consists of a shell made through stereolithography using a polycarbonate-like, bio-compatible material (used for dental replacement: ‘Accura ClearVue (SLA)’, 3D-Systems, Inc., Rock Hill, SA, USA) with a thickness of 4 mm and an internal volume of 5.3 L, (Figure 2A–C), along with a large axial circular opening below the neck to allow placement of the icEEG electrode and filling with gel. The shell’s material has the following physical properties: viscosity of 235–260 cps (in 30 °C), critical exposure of 9.0 mJ/cm^2^, and liquid density of 1.10 g/cm^3^ (at 25 °C). The external surface of the shell has 22, 2 mm-deep disk-shaped indentations positioned in accordance with the international 10–20 system for scalp EEG [57].

Following the placement of the icEEG electrodes and temperature probes inside the head part of the phantom (see the following sections for further details), it was filled with an agar semi-solid gel made by mixing heated tap water, 4% Agar (Sigma–Aldrich, Merck, Germany), 0.5% NaCl, and 4 mL/litre of a preservative agent [22,58]. A lab stirrer was used to provide vibrations to ensure that the whole space was filled while minimising bubbles or voids between the electrodes (a semi-solid gel was used in order to hold the implanted electrodes in position during the phantom manipulations and prevent leakage once the head is placed in a supine position inside the QS-ASTM-II phantom).

### 2.2. Electrode Description and Placement

#### 2.2.1. IcEEG Electrodes—Experiment 1

Three depth electrodes, one strip electrode, and one grid electrode (all made by Ad-Tech Medical Instrument, Oak Creek, WI, USA) were placed inside the head phantom to represent a representative implantation in our centre (Figure 2C), in line with our previous safety investigations [6,42]. The depth electrodes were of two models, one of type ‘SD-8PX’ (8 platinum contacts with 10 mm spacing, total length: 380 mm) and two ‘SD-6PX’ (6 platinum contacts with 10 mm spacing, total length: 370 mm). The strip electrode was ‘T-WS-6PX’ (6 platinum–iridium contacts with 10 mm spacing, total length: 445 mm). The grid electrode was of type ‘T-WS-48PX’ (6 × 8 platinum–iridium contacts with 10 mm spacing, total length: 455 mm). The contacts of the strip and grid are 4 mm diameter disks embedded in the silicon housing, exposed on one side by 2.3 mm. The wires of the depths, strip, and grid electrodes are made of nickel–chromium and isolated from each other, with each wire connected to one contact on one side and contained inside a polyurethane tubing on the other side.

For *Experiment 1*, the following depth electrodes were positioned inside the empty head phantom similarly to our previous experiments [6,42] using a frameless technique [59,60] (Figure 2C): the three depth electrodes along the lateral trajectories: two 8-contact depths: right temporal anterior (‘*R*’) and left temporal posterior (‘*LP*’); one 6-contact depth: left temporal anterior (‘*LA*’). The trajectories were planned to mimic that of a bi-temporal bi-hippocampal implantation. As is conventional in the stereo-encephalographic (SEEG) approach to icEEG electrode implantation, the trajectories were first aligned to the pre-defined plan so that drilling could be performed. A bolt was then screwed into the phantom along the drilled trajectory. The electrodes were then passed through the inner channel of the bolt into the phantom. Following this, the electrodes were held in position with cotton threads attached to the ends of the depth electrodes and extended and attached to the side of the head contralateral to the insertion point.

A 6-contact subdural strip (‘*S*’) was placed approximately over the left parietal–frontal convexity (at the superior region of the head) and a 6 × 8-contact subdural grid (‘*G*’) was placed on the inner concavity of the phantom skull to mimic a fronto–parietal grid over the right frontal convexity. These were fixed to the phantom’s inner surface using glue (see Figure 2). The phantom was then filled as described in the preceding section.

**Figure 3:**
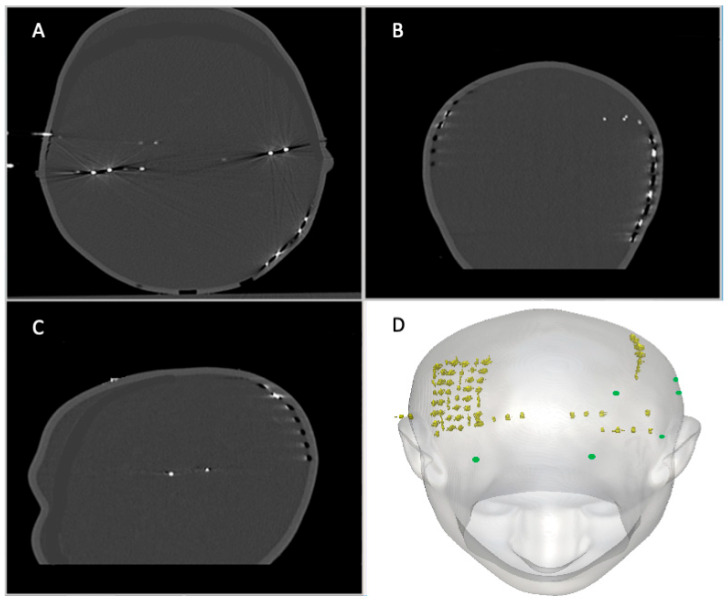
**Images of the phantom with subdermal scalp and icEEG electrodes in place**: (**A**–**C**) are CT images of the realistically shaped head phantom with the implanted icEEG electrodes and Ives subdermal wire electrodes. SWEs are fixed in the following locations: FP1, T5, F7, FP2, F3, and T3, which follows the 10–20 international scalp placement system. (**A**): Axial view of the skull. (**B**): Coronal view of the skull. (**C**): Sagittal view of the skull. (**D**): Top 3D view of the head shell with the icEEG electrodes in yellow and the Ives subdermal wire electrode positions in green.

In accordance with our icEEG-fMRI protocol [6,61], all electrode leads were gathered in two bundles at the top (superior aspect) of the head part of the phantom, with one bundle on the right comprising *R* and *G* leads and the second bundle on the left containing *LA*, *LP*, and *S* leads. All the leads were then connected to the 90 cm extension cables, which were connected to an input box. See Figure 3A–D for the CT images of the 3D head shell.

#### 2.2.2. Subdermal Scalp EEG Electrodes—Experiment 2

In this experiment, in addition to the icEEG electrodes placed for *Experiment 1*, six subdermal wire electrodes (SWE; Ives EEG Solutions, Newburyport, MA, USA) were placed on the surface of the head part of the phantom. The number of electrodes chosen reflects our choice to limit the scalp element of the EEG recording for future application to six electrodes placed strategically to detect specific EEG discharges, which were localised in advance of the scalp-icEEG-fMRI acquisition from a previously recorded scalp EEG.

**Figure 4:**
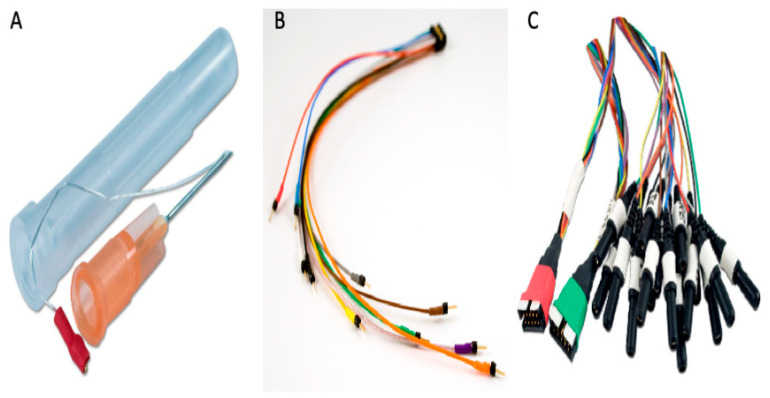
**Illustration of the Ives subdermal scalp electrode**: (**A**): Subdermal wire electrodes (SWEs). (**B**): Subdermal wire electrode adaptor (SWE-ADT). (**C**): Subdermal wire electrode harness (SWE-HAR). Adapted from https://www.iveseegsolutions.com/swe (accessed on 13 January 2025).

Each electrode consists of three components: (1) the subdermal wire electrode itself (‘SWE’) (Figure 4A), which is composed of a subdermal stainless steel needle connected to a wire made of pure silver and a recording tip (length: 3 mm); (2) an electrode adaptor (‘SWE-ADT’) (Figure 4B), which contains two terminals: one terminal with several pin-like color-coded endings that can be attached with the SWE at one end and another terminal that can be attached with the third component, namely, (3) an electrode harness (‘SWE-HAR’) (Figure 4C) that connects the ‘SWE’ and ‘SWE-ADT’ to an EEG input box.

The subdermal EEG electrodes were placed by insertion into a small amount of modelling clay in the aforementioned disk-shaped indentations at the following EEG electrode positions: F3, F7, FP1, FP2, T3, and T5 (see Figure 3D). These were chosen based on pilot data (unpublished) and other published research indicating that they are the locations of maximum heating [22,23,26,28,31,62]. The leads of the scalp electrodes were placed at the top of the phantom along with those of the icEEG electrodes.

### 2.3. Temperature Probes

The temperature measurements were performed using the following fibre-optic sensors: model T1C-10-PP05 and model T1C-10-B05, Neoptix, Québec, QC, Canada; sampling rate = 1 Hz, resolution = 0.1 °C), connected to four-channel signal conditioners (SCs): SC-1 and SC-2 (Neoptix ReFlex—Neoptix Inc., Canada; sampling rate = 1 Hz; resolution = 0.1 °C); model OTG-M280, connected to SC-3 (Model TMS-G4-10-100ST-M2; OpSens-TempSens, OpSens Solutions Inc., Québec, QC, Canada; sampling rate = 50 Hz, resolution = 0.1 °C).

#### 2.3.1. Experiment 1: Description

Seven fibre-optic sensors were placed in close physical contact with the following electrode contacts (Figure 2C) and connected to signal conditioners SC-1 and SC-2: (1) *R* contact #1 (*R-1*): the probe was placed parallel and attached to the to the most distal electrode contact (#1); (2) *R* contact #5 (*R-5*): the probe was placed parallel and attached to electrode contact #5; (3) *LA* contact #1 (*LA-1*): the probe was placed parallel and attached to the most distal electrode contact (#1); (4) *LA* contact #5 (*LA-5*): the probe was placed parallel and attached to electrode contact #5; (5) *LP* contact #1 (*LP-1*): the probe was placed parallel and attached to the most distal electrode contact (#1); (6) *S* contact #6 (*S-6*) the probe was placed parallel and attached to the middle of electrode contact #6; (7) *G* contact #48 (*G-48*): the probe was placed in parallel and attached to the middle of electrode contact #48. The temperature probes were secured to the electrodes using cotton thread, taking great care to ensure that the contact between the temperature probe and the electrode was as close as possible [63].

A temperature probe was placed in the body of the phantom (*Ref*) around the right shoulder area and connected to SC-3. However, due to a technical problem, the temperature at *Ref* in *Experiment 1* was monitored (but not recorded) and the maximum value noted.

#### 2.3.2. Experiment 2: Description

The temperature was recorded at twelve locations. Eight sensors were placed in contact with the following electrodes and connected to SC-1 and SC-2: Seven at icEEG electrode contacts *LA-1*, *LA-5*, *LP-1*, *S-6*, *R-1*, *R-5,* and *G-48*; one was placed at scalp electrode *F3* (the sensor at *LA-5* stopped functioning after *Experiment 1*). Four temperature probes were placed in contact with the following scalp electrodes and connected to SC-3: *FP1*, *T5*, *F7*, and *FP2*). A temperature probe was placed in the body of the phantom (*Ref*) and connected to SC-1.

### 2.4. RF Exposure Protocol and Temperature Measurements

*Experiments 1* and *2* were performed one week apart inside a Siemens 1.5 T Avanto (Siemens AG, Erlangen, Germany: software VB17A) MRI scanner. We used the scanner’s standard Siemens transmit RF whole-body coil only. A high-SAR turbo spin echo (TSE) sequence was used with the following sequence parameters: TR 2850 ms; TE 92 ms; bandwidth: 125 Hz/pixel; FOV 30 × 30 cm; 35% PE oversampling; matrix 384 × 346; 13 slices; slice thickness (ST) 2.5 mm; slice spacing (SS) 1.25 mm; number of excitations: 4, concatenations 1, turbo factor 15, and a duration of 6 min 9 s. Across temperature measurements, the scanner-reported head-average SAR values were in the range of (2.9–3.1) W/kg and B_1+RMS_ in the range of (4.1–4.3) µT. This high-SAR sequence was used to create a worst-case heating scenario.

For *Experiment 2*, an additional, low-SAR EPI sequence was used with the following scan parameters: TR 4480 ms; TE 50 ms; bandwidth: 2298 Hz/pixel; FOV 19.2 × 19.2 cm; 0% PE oversampling; matrix 64 × 64; 49 slices; slice thickness (ST) 2.0 mm; slice spacing (SS) 1 mm; number of excitations 1, concatenations 1, and duration of 6 min 4 s. The scanner-reported head-average SAR values were 0.1 W/kg and B_1+RMS_ 0.9 µT.

In *Experiment 1*, two 6-min series of temperature measurements were performed while the high-SAR sequence was run. The interval between the two series was 10 min to allow the temperature to return to baseline. Immediately after *Experiment 1*, the phantom was removed from the scanner and stored in a room kept at around 20 °C until *Experiment 2*. The scalp electrodes and additional temperature probes were placed on the phantom during this period.

In *Experiment 2*, two 6-min series of temperature measurements were performed while the high-SAR sequence was run, and a third series was performed with the low-SAR sequence. The interval between the series was 10 min.

## 3. Results

### 3.1. Experiment 1

**Figure 5:**
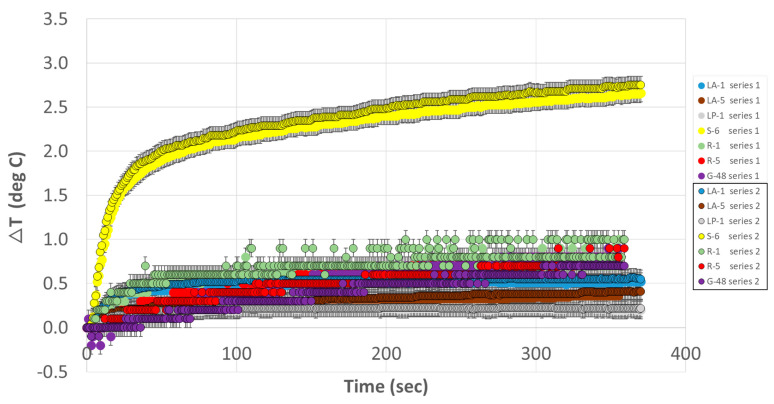
**Temperature changes observed in Experiment 1**. Series #1 and 2. Temperature measurement locations indicated as icEEG electrode contact labels: LA-1, LA-5, LP-1, S-6, R-1, R-5, and G-48. The error bars represent the thermometer’s +/−0.1 °C resolution. A similar repeatability pattern was observed for Experiment 2, Series #1 and 2.

Figure 5 shows the time course of temperature changes during the first of this experiment’s two series. The maximum (peak over the 6-min exposures) temperature increases are presented in Table 1. The maximum temperature change across the two series was +2.8 °C at *S-6*, and the maximum inter-series peak temperature change variation was <0.2 °C, at *G-48* (mean absolute inter-series variation: 0.1 °C).

### 3.2. Experiment 2

The maximum temperature increases are presented in Table 2. For the high-SAR TSE sequence, the maximum temperature changes for the icEEG electrodes were in the range [+0.1–+2.1 °C]. The observed temperature changes (averaged over the two series) did not significantly deviate from those for *Experiment 1,* with an average of +0.15 °C (*p* = 0.2, two-sided paired *t*-test) and a maximum inter-experiment deviation of +0.85 °C (at *S-6*).

For the subdermal scalp electrodes, the maximum temperature changes were in the following range: [+0.1–+0.6 °C].

For the low-SAR sequence, there was no significant (i.e., greater than instrumental precision of 0.1 °C) temperature increases detected for the icEEG electrodes; for the subdermal scalp electrodes, the maximum temperature change across the two series was +0.7 °C (location *T5*), and the maximum inter-series peak temperature change variation was 0.3 °C, also at *T5*.

## 4. Discussion

In this study, we aimed to evaluate the potential additional risks of RF-induced heating associated with adding subdermal scalp EEG electrodes to a set of icEEG electrodes in order to determine the feasibility of performing simultaneous scalp EEG and icEEG recordings during functional MRI scanning, following our recent work on our 1.5 T scanner’s body RF transmit coil for fMRI in the presence of icEEG electrodes [41]. We achieved this by comparing the heating patterns in the immediate vicinity of EEG electrodes for two configurations: a baseline, which consists of icEEG electrodes placed in a test phantom, and a test configuration, obtained by adding six subdermal scalp EEG electrodes to the baseline one, in a configuration that is representative of the type of experimental setup we envisage for combined scalp-icEEG-fMRI. In these configurations, the addition of subdermal scalp electrodes did not result in a significant change in the overall high-SAR RF-induced temperature changes in the vicinity of the icEEG electrodes. For the EPI sequence RF exposure, the measured temperature increases in the vicinity of the icEEG electrodes were low, consistent with our previous observations [32,41,61].

The baseline icEEG electrode configuration and cable path were chosen to match the scenario we previously investigated [6,42,61]; in particular, the importance of careful and systematic cable path management to help reduce the health risks, specifically by bundling and routing them at the top of the head/phantom along the scanner’s Z-axis (this configuration also offers EEG data quality benefits ([64]; see also [65]). Following this guidance allowed us to subsequently perform concurrent icEEG-fMRI data acquisitions successfully in twenty patients with data at our centre, without any observation or reporting of adverse effects [33,66,67,68] and with direct evidence of the absence of significant adverse effects [43]. For the test configuration used here, the small number (six) of scalp electrodes added reflects our wish to perform combined scalp-icEEG-fMRI acquisitions in a targeted (rather than exploratory) manner, which will be based on the results of prior scalp EEG recordings and, therefore, we intend to use only six scalp electrodes in human subjects. This approach is mostly motivated by the logistical problem of adding scalp EEG electrodes immediately after the implantation of the icEEG ones, in the operating theatre, before the application of a head bandage and subsequent maintenance; limiting their number will greatly facilitate this process. In addition, the primary scientific objective of the scalp-icEEG-fMRI experiments we propose to undertake is the study of the relationship between BOLD fluctuations and specific focal pathological (epileptiform) electrophysiological discharges that will have been seen on previous scalp EEG recordings. The focal nature of these patterns means that they will be most clearly visible on a small number of electrode positions, which we will use to allow their detection during scalp-icEEG-fMRI. Furthermore, the use of a small number of scalp electrodes also has the advantage of simplifying the safety issues. We restricted our experiments to circuit configurations, consistent with our own icEEG-fMRI protocol [61], namely, connecting leads placed along the scanner’s central (*Z*) axis and connected to the input box.

In accordance with common practice for this type of test, we used high-SAR exposures to reflect the recommended safety guidelines [69], effectively as a ‘worst case’ scenario, and low-SAR exposures to reflect the actual proposed experimental conditions for the application of scalp-icEEG-fMRI in humans better, in accordance with our established, strict scanning protocol [61]. In both scenarios, the phantom experiments performed according to the ASTM protocol, as in this study, offer an additional safety margin due to the lack of blood flow and tissue perfusion, which act as heat sinks in living beings, meaning that the temperature increases observed here are higher than would occur in a real, human body-loading scenario [70].

In summary, our results demonstrated that the addition of the scalp electrodes had a moderate to small effect on the observed heating in the vicinity of the icEEG electrode (compared to baseline), and that the heating for a low-SAR exposure was well within the accepted safety guidelines and in the immediate vicinity of all scalp and intracranial electrode locations. Specifically, for the high-SAR exposure, the maximum temperature change across the two series was +2.8 °C, at subdural strip location *S-6*. We note that this location, which was not recorded in the previous safety studies, was partly due to the limited number of temperature probes we had access to at the time [6,42]. For the high-SAR exposure, the temperature increases at the other icEEG contacts did not exceed +1 °C. Furthermore, repeated measurements at high SAR demonstrated excellent reproducibility with a maximum inter-series peak temperature change variation of 0.2 °C. Adding a set of six scalp electrodes in *Experiment 2* resulted in a difference in temperature increase of +0.85 °C or less across the icEEG electrodes. For the low-SAR fMRI-type gradient-echo EPI sequence, the peak temperature increases were ≤+0.7 °C, with the maximum heating observed at a scalp electrode location, and the heating at all icEEG locations was within measurement precision (±0.1 °C).

The range of peak temperature increases measured in the vicinity of the icEEG electrodes without scalp electrodes (Experiment 1, high-SAR exposures) in this work is similar to those observed in our previous investigations of heating in the vicinity of icEEG electrodes using the head transmit RF coil [6,42]. The results of our repeated observations of the heating around the icEEG electrodes suggest that differences in phantom and wire positioning (that occurred between our two experiments, despite our best efforts to minimise them) are probably as important a source of heating variation as the addition of the scalp electrodes.

Concerning the spatial heating pattern, we observed that the heating at the most distal depth electrode contacts (see Table 1 and Table 2) is higher than the heating at contact # 5 (*R-5* and *LA-5*) of the same electrode, which is consistent with the findings of the electromagnetic computational simulations of heating near wires [71] and depth electrodes [40,42]. This effect results from the coupling between the electrical component of the RF field and linear metallic implants, which leads to alteration of the electric field in the vicinity of the implant. In particular, this can result in considerable localised field intensity increases at the tips of the implants associated with the field’s tangential component due to charge accumulation (electric displacement field) at sharp conductivity boundaries [72], as demonstrated empirically for deep brain stimulation electrodes [53,54].

With regards to heating in the vicinity of scalp electrodes, previous studies recorded maximum temperature increases at 3 T of about 4 °C at an ECG electrode placed at the clavicle area inside a head transmit coil and about 4 °C at T8 and about 3.5 °C at Cz inside a body transmit coil [62], as well as 4.1 °C at FP2, 3.2 °C at T7, 1.4 °C at FP1, and 0.3 °C at T8 for phantom measurements, and 2.1 °C at F4, 1.1 °C at CPz, and 1 °C at FP1 and FT8 for human volunteer measurements [28]. At 7 T, previous studies recorded maximum temperature increases of 3.81 °C and 1.15 °C around FP1 and Cz, respectively, inside a TEM T/R head coil [31], 3.64 °C at Cz paste, 0.9 °C at 5 mm around FP1, and 0.15 °C at the centre for the InkCap electrode and 6.6 °C at Cz paste, 0.89 °C at 5 mm around FP1, and 0.19 °C at the centre for the Quick Cap electrode [26], and 0.4 °C, 0.25 °C, and 0.2 °C at FP1, FP2, and Cz, respectively [22]. In addition, at 7 T, safety tests of temperature measurements showed increases of ≤0.7 °C around scalp EEG electrodes and 6.5 °C at the EEG amplifier [23].

### 4.1. Methodological Considerations

A wide range of phantoms have been used in previous studies on the safety of RF-induced heating in the vicinity of electrodes (scalp or invasive EEG, or for deep brain stimulation) in MRI (all filed with a solution or gel, except for the animal specimens), including two bags [36], plastic or acrylic spheres or hemispheres [21,22,27,32,73], an acrylic sphere and torso (separate) [38], a porcine cadaver head [74], acrylic cylinders [75,76,77,78], a realistically shaped conductive solid gel head [26,29,31,79], an acrylic square-shaped head and torso (ASTM; [6,37,39,42,49,50,51,52,53,54,55]), rabbit cadaver [37], a watermelon [30], and a synthetic skull and torso (connected; [80]). None of those satisfied the need to accommodate both intracranial and scalp EEG electrodes in a realistic fashion. Therefore, we developed a new phantom for this work that allows realistic representations of electrode implantations combined with adequate body coil loading and followed the prescribed gel recipe to ensure compliance with the ASTM guidance, as validated in [81].

This work benefited from greater spatial sampling of the temperature changes than our previous experiments on heating in the vicinity of icEEG electrodes, due to the availability of 12 temperature probes compared to four [6,42,61] and eight [22,62]. We used this extra flexibility to obtain a good set of repeat measurements across the two experiments while maximising spatial sampling. An important consideration in this regard was the procedure for placing the icEEG electrodes and temperature probes, and filling the phantom with gel. Initial tests demonstrated that it would be extremely difficult to imitate the surgical process of inserting icEEG electrodes inside a gel-filled phantom, partly due to the need to position the temperature probes in direct contact with the chosen electrode contacts [63]. As in our previous work, we tied each temperature probe to the electrodes with the cotton thread, resulting in a combined (electrode + temperature probe + thread) assembly that cannot be inserted in a gel-filled phantom using surgical instruments, or in any matter that we could find that would not cause damage to the gel with large air pockets and to the assemblies themselves. Therefore, we chose to position the (electrode + temperature probe + thread) assemblies within the phantom prior to filling; the subdural grids and strips were glued to the phantom’s inner surface to ensure that they remained in position during the gel filling process. Once the saline had gelled, removing or adding temperature probes became extremely difficult. Therefore, all icEEG electrodes and associated temperature probes were fixed for the two experiments, and some of the available temperature probes were unused in *Experiment 1* because they were necessary for *Experiment 2*.

Finally, our recent work, using the same temperature measurement technology as in this work, showed a degree of reproducibility [81] corresponding to a detection threshold of the order of 0.5 °C [41].

### 4.2. Choice of Subdermal Scalp Electrodes

In accordance with our current practice, the proposed experiments will be performed at the end of the clinical invasive EEG investigations, which can last up to two weeks. In this regard, the chosen subdermal electrodes offer a crucial advantage over standard disk and gel scalp electrodes, namely, their capability to maintain recording quality over weeks without maintenance [44] in a scenario in which they are placed immediately after the surgical insertion of the icEEG electrodes and covered by the standard surgical bandage. This also limits the risk of infection.

### 4.3. Limitations of This Work

In addition to the previously mentioned issue of the limited spatial sampling of the heating patterns observed using temperature probes, the number of electrode configurations tested is limited. While these were chosen to be representative and realistic representations of implantations used in our centre’s clinical practice, they are a sample from part of a potentially wide range of implantation configurations, and, therefore, the generalisability of our findings may be questioned [20]. Two considerations are worth mentioning: First, generalisability may be taken to encompass the concept of worst-case testing scenario as featured in the relevant ASTM standard [55] with the additional, more explicit, consideration of the more general conditions within which the finding could be interpreted and applied (in scanning humans). Second, the RF-induced heating tests presented here build directly on our previous tests for evaluating the risks associated with icEEG-fMRI [6,38,42] and scalp EEG-fMRI [22,23,26,28,31,62] separately, both of which were performed based on the recommended standard [55] where applicable. With regards to the first point, as in our previous publications, we hereby stress that our findings are directly relevant solely for our EEG and MRI data acquisition methodologies, in particular the MRI scanner type, including and especially the particular RF transmit coil used, and the configuration of icEEG and EEG electrodes, leads, and other elements of the recording chain [61]. Therefore, while the tests presented here were inspired by the ASTM standard, their aim is not to obtain approval from regulatory bodies for commercial purposes, i.e., as grounds for an MR-conditional statement to allow scalp-icEEG-fMRI to be performed outside the confines of our centre. Hence, our recommendation remains that a thorough local risk assessment is required for a centre wishing to apply these findings to their local setting. With regards to the second point, we would like to point to the lack of any adverse event reported for either icEEG-fMRI (20 datasets acquired to date at our centre; see also [43] for direct evidence in a small sample) or scalp EEG-fMRI (more than 400 datasets acquired to date at 1.5 and 3 T, both using our locally developed and tested EEG electrodes and amplification equipment, and commercial EEG caps and amplifiers) at our centre as supporting our general approach to safety testing and data acquisition protocols. In addition, we note that, as a result of our safety investigations and those of others [36,37,38,40,41,61]—often conducted using much simpler phantoms—icEEG-fMRI experiments have now been performed in a total of more than 100 patients to date in three centres [82,83,84,85], with no reports of adverse events.

The applicability of the conclusions reached in this study is limited to the type of MRI instrument tested here, namely, a Siemens 1.5 T Avanto. In line with the gradual and systematic approach to implementing combined EEG and fMRI data acquisitions that we have followed since the start of our work in this area [6,32,41] (see [86] for an overview), any application of concur-rent scalp-icEEG-fMRI acquisitions on a significantly different instrument, such as a 3T scanner, would require care-ful consideration of all safety aspects and therefore additional tests similar to those performed here. The same cautionary principle would apply to any proposed substantial change to the methodology related to the EEG element, such as coverage using more than six scalp electrodes for concurrent scalp-icEEG-fMRI acquisitions.

Finally, well-validated computational electromagnetic simulations may be helpful in this regard by providing estimates of the location of hot spots to be validated experimentally. While this approach is attractive in principle, our own limited experience in this field demonstrates that the computational demands are considerable, given the complexity (fine detail of the small-scale elements) of the electrodes we use.

## 5. Conclusions

In conclusion, this study demonstrates that performing fMRI using the body RF-transmit coil on our whole-body 1.5 T MRI scanner, in the presence of a small number of subdermal scalp EEG electrodes combined with icEEG electrodes, does not lead to significant additional heating compared to the icEEG-fMRI-only scenario in the specific configurations tested here. As per our previous work, avoiding high-SAR sequences is a particularly important precaution, along with careful, controlled EEG lead placement. Additionally, our methods and results provide valuable information to assist other researchers in conducting risk evaluations and implementing scalp-icEEG-fMRI in their facilities.

## Figures and Tables

**Table 1:** **Maximum temperature increases for Experiment 1**. High-SAR exposure with one repetition (Series 1 and 2). The maximum increase was observed at the strip electrode (S-6). The temperature measurement precision is 0.1 °C.

Series #		Location	Maximum Temperature Increase (°C)						
	MRI Sequence		icEEG Electrode Contact Label						
			LA-1	LA-5	LP-1	S-6	R-1	R-5	G-48
1	TSE		0.5	0.4	0.2	2.7	1.0	0.8	0.9
2	TSE		0.6	0.4	0.2	2.8	1.0	0.9	0.7

**Table 2:** **Maximum temperature increases for Experiment 2**. Two high-SAR exposures and one low-SAR exposure, performed one week after Experiment 1. As in Experiment 1, the peak temperature increases were observed at the strip electrode (S-6); on the whole, the peak increases were lower at the scalp electrode locations. The temperature measurement precision is 0.1 °C.

Series #		Location	Maximum Temperature Increase (°C)										
	MRI Sequence		icEEG Electrode Contact Label						Scalp Electrode Contact Label				
			LA-1	LP-1	S-6	R-1	R-5	G-48	F3	FP1	T5	F7	FP2
1	TSE		0.5	0.1	2.1	1.3	1.2	0.3	0.2	0.2	0.4	0.5	0.6
2	TSE		0.6	0.1	1.7	1.2	0.9	0.4	0.2	0.3	0.4	0.3	0.6
3	EPI		<0.1	<0.1	<0.1	<0.1	<0.1	<0.1	<0.1	0.4	0.7	0.3	0.5

## Data Availability

On request by contacting louis.lemieux@ucl.ac.uk.
